# Modeling early treatment response in AML from cell-free tumor DNA

**DOI:** 10.1016/j.isci.2023.108271

**Published:** 2023-10-19

**Authors:** Dantong Wang, Christian Rausch, Simon A. Buerger, Sebastian Tschuri, Maja Rothenberg-Thurley, Melanie Schulz, Jan Hasenauer, Frank Ziemann, Klaus H. Metzeler, Carsten Marr

**Affiliations:** 1 Institute of AI for Health, Helmholtz Zentrum München - German Research Center for Environmental Health, Neuherberg 85764, Germany; 2 Center for Mathematics, Technische Universität München, Garching 85748, Germany; 3 Laboratory for Leukemia Diagnostics, Department of Medicine III, University Hospital (LMU), Munich, Germany; 4 German Cancer Consortium (DKTK), partner sites Munich/Dresden, Germany; 5 Computational Health Center, Helmholtz Zentrum München - German Research Center for Environmental Health, Neuherberg 85764, Germany; 6 Faculty of Mathematics and Natural Sciences, Rheinische Friedrich-Wilhelms-Universität Bonn, 53115 Bonn, Germany; 7 Department of Hematology and Cell Therapy, University Hospital Leipzig (UHL) 04103, Germany

**Keywords:** Disease, Biological sciences

## Abstract

Monitoring disease response after intensive chemotherapy for acute myeloid leukemia (AML) currently requires invasive bone marrow biopsies, imposing a significant burden on patients. In contrast, cell-free tumor DNA (ctDNA) in peripheral blood, carrying tumor-specific mutations, offers a less-invasive assessment of residual disease. However, the relationship between ctDNA levels and bone marrow blast kinetics remains unclear. We explored this in 10 AML patients with *NPM1* and *IDH2* mutations undergoing initial chemotherapy. Comparison of mathematical mixed-effect models showed that (1) inclusion of blast cell death in the bone marrow, (2) transition of ctDNA to peripheral blood, and (3) ctDNA decay in peripheral blood describes kinetics of blast cells and ctDNA best. The fitted model allows prediction of residual bone marrow blast content from ctDNA, and its scaling factor, representing clonal heterogeneity, correlates with relapse risk. Our study provides precise insights into blast and ctDNA kinetics, offering novel avenues for AML disease monitoring.

## Highlights

• Mixed effect model fits bone marrow blasts to ctDNA kinetics on systems level
• We predict bone marrow blast counts during treatment from peripheral blood ctDNA
• Model’s scaling factor indicates patient with increased relapse risk

## Graphical abstract

**Figure:**
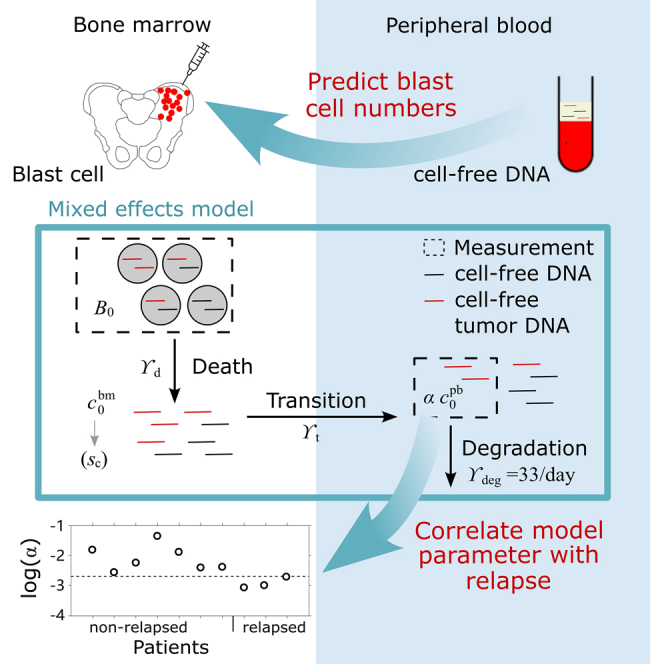

## Introduction

Acute myeloid leukemia (AML) is the most common acute leukemia in adults.^1^ In patients with AML, cell differentiation stops at the myeloblast stage;^2^^,^^3^ leukemic myeloblasts fill the bone marrow niche, displace normal hematopoiesis, and lead to severe illness. Leukemic blasts harbor mutations that distinguish them from benign myeloblasts. Analysis of driver mutations is part of the routine diagnostics to assess risk stratification in AML. Most common mutations are found in *FLT3* (39%), *NPM1* (33%), *DNMT3A* (31%), *NRAS* (22%), *RUNX1* (15%), *TET2* (15%), and *IDH2* (14%).^4^ Initial diagnosis of AML includes a bone marrow aspiration to determine the percentage of blast cells in the bone marrow. The current standard of care for fit AML patients involves an initial phase of intensive chemotherapy followed by post-remission treatment including additional chemotherapy and/or allogeneic stem cell transplantation.^5^ Within 16 to 21 days after beginning of intensive chemotherapy, a second bone marrow sample is often taken to evaluate the reduction in the amount of blast cells (blast clearance). Additional follow-up bone marrow biopsies are needed for disease monitoring during and after post-remission therapy, and are associated with considerable patient discomfort.

To reduce the burden on the patient, time-resolved monitoring of cell-free tumor DNA (ctDNA) has been proposed.^6^ ctDNA consists of small DNA fragments found in blood plasma of cancer patients and can be used to detect tumor mutations.^7^^,^^8^ ctDNA is defined as a small fraction of cell-free DNA (cfDNA) which refers to all circulating DNA in the bloodstream. cfDNA occurs naturally in all individuals, regardless of health status, and is released from dying cells and found to be increased in states of increased cell turnover (e.g., acute trauma, exercise, infection).^9^

Decreasing ctDNA levels during the initial phase of induction chemotherapy are a promising marker for early assessment of treatment response and a prognostic tool in AML patients.^10^^,^^11^^,^^12^ However, it is still not clear how the ctDNA detected in peripheral blood is related to the percentage of blast cells in the bone marrow. This is important because elimination of leukemic blasts in the bone marrow after initial chemotherapy is a prerequisite for achieving complete remission, an important treatment milestone with favorable prognosis for AML patients. A mathematical model can link these two variables in measured time series.

Biological processes are usually described by models with ordinary differential equations (ODE). The parameters of such models are usually the initial values of the model species and the rate constants of the described reactions.^13^ Fitting ODE models to clinical time series can estimate unknown parameters of the model and unmeasured states. For heterogeneous cell or patient populations, model parameters are often assumed to be distributed, resulting in nonlinear mixed effects models.^14^^,^^15^^,^^16^^,^^17^ Mixed effects models assume that the parameters are composed of fixed and random effects. The fixed effects are the same for the entire patient population, whereas the random effects are different for each individual patient and follow, for example, normal or log-normal distributions. In mixed effects models, the unknown parameters are usually estimated by maximizing a likelihood function, which can be understood as the similarity between the model simulation and the measured data. For heterogeneous diseases such as AML,^18^^,^^19^ the use of mixed effects models is particularly relevant. In our case, the initial blast count and peripheral blood ctDNA concentration differ between patients due to individual effects, such as the time of diagnosis. Therefore, mixed-effects models were used to describe the variability from patient to patient and to estimate the population parameters for all patients simultaneously.

In this work, we studied the percentage of bone marrow blast cells and ctDNA concentration in peripheral blood from 10 AML patients undergoing initial chemotherapy.^20^ We focus our analysis on *NPM1*, an established measurable residual disease (MRD) by recent ELN recommendation,^21^ and on *IDH2*, an often mutated gene we have previously shown to be used in MRD follow-up of AML patients.^20^ In contrast to these two genes, other frequently mutated genes such as *DNMT3a, TET2*, and *ASXL1* have been shown to be not suitable for MRD assessment as they are consistent with premalignant clonal hematopoiesis^22^ and persist in long-term remission, not contributing to an increased relapse risk. Therefore these three mutations are not recommended for MRD.^21^

We compared 3 hypotheses for ctDNA kinetics using ODE model implementation and parameter fitting for a mixed-effects model. We then proved that the best-fitting model was structurally identifiable and able to predict the percentage of blast cells in bone marrow from peripheral blood ctDNA data. A model-inherent scaling factor representing heterogeneity of AML-related mutations correlated with relapse risk.

## Results

### ctDNA and bone marrow blast cell measurements of 10 AML patients during chemotherapeutic treatment

**Figure 1:**
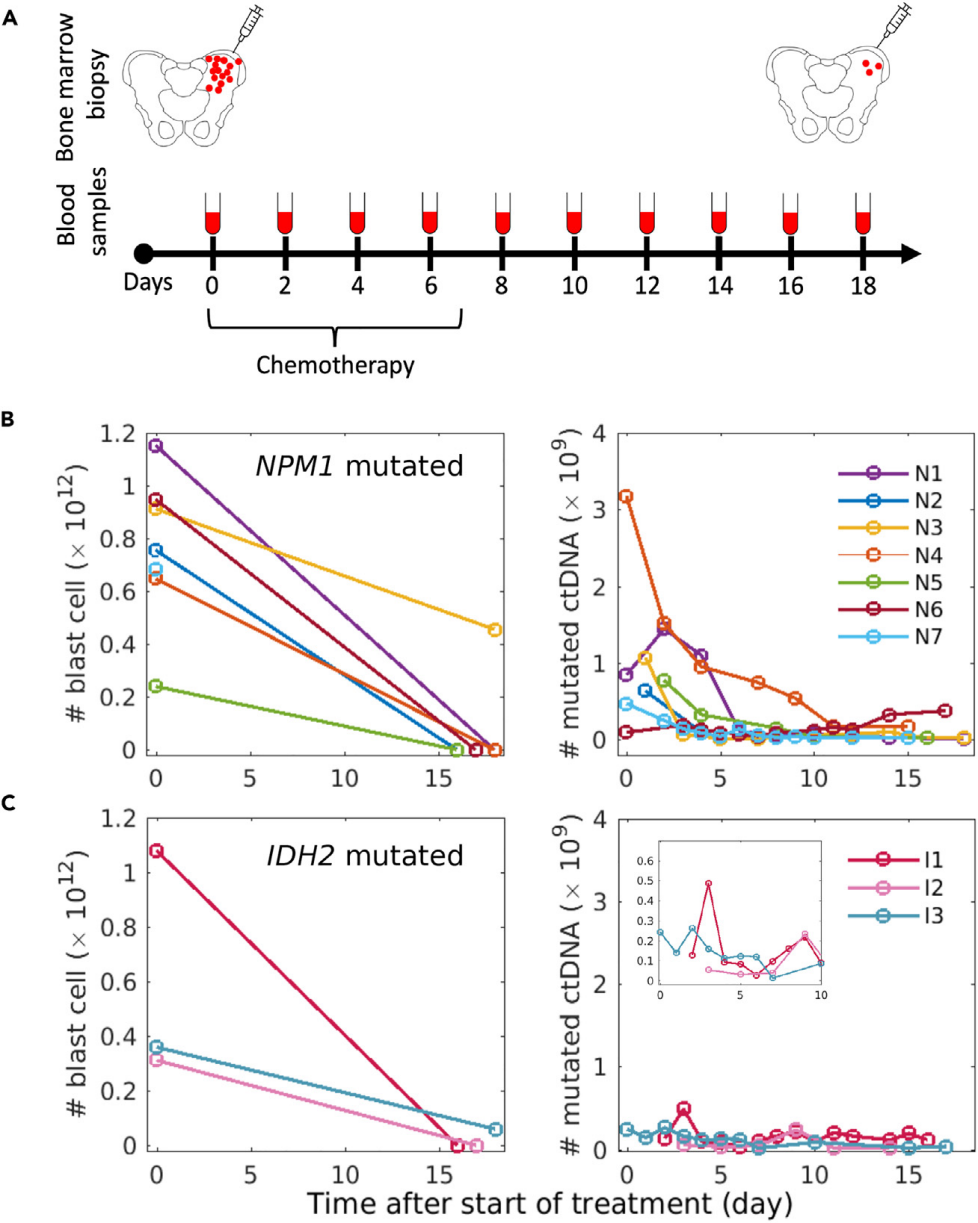
Blast cell and ctDNA kinetics of 10 AML patients under chemotherapy treatment (A) Overview of sample collection. Bone marrow aspiration is taken at initial diagnosis and 16 to 21 days after start of chemotherapy, to assess leukemic blast count (red dots) within the bone marrow. Peripheral blood, for measuring ctDNA kinetics, was collected at least every second day during the first 21 days after beginning of treatment. (B) Absolute number of bone marrow blast cells and ctDNA for seven *NPM1* mutated AML patients. (C) Absolute number of bone marrow blast cells and ctDNA for three *IDH2* mutated AML patients.

We used data from 10 AML patients treated at the LMU department of hematology, as previously described.^20^ Blast cell percentages in bone marrow were measured for all patients at initial diagnosis and 16–18 days after beginning of induction therapy (Table 1; Figure 1A). The ctDNA concentration for two recurrent AML-related mutations, affecting the *NPM1* and *IDH2* genes, was measured between 2 and 14 times (mean = 8.7, std = 3.7) during the first 18 days after initiation of induction chemotherapy. During model fitting, we scaled measurements to absolute blast cell numbers and ctDNA numbers in the whole body instead of blast cell percentage and ctDNA concentration (Figures 1B and 1C). For this purpose, we first calculated the patient specific blood volume using body weight and height as described by Nadler et al.^23^ Then, we assumed the nucleated cell number in the bone marrow to be $1.2\cdot 10^{12}$.^24^ Details of the underlying assumptions can be found in the Methods.

**Table 1:** Clinical data of *NPM1* and *IDH2* mutated patients (see for detailed patient characteristics)

Patient ID^20^	Age	Gender	Blood volume in L	Mutation	Relapse	Risk group *(ELN 2017)*	# ctDNA measurements	# blast cell measurements
N1	28	Female	4.0	*NPM1*	No	Intermediate	9	2
N2	36	Female	4.7	*NPM1*	No	Favorable	2	2
N3	46	Female	3.6	*NPM1*	No	Intermediate	9	2
N4	52	Female	4.5	*NPM1*	No	Favorable	7	2
N5	59	Male	5.9	*NPM1*	No	Favorable	5	2
N6	60	Female	4.2	*NPM1*	No	Favorable	12	2
N7	67	Male	5.1	*NPM1*	Yes	Favorable	12	1∗
I1	55	Female	4.6	*IDH2*	No	Intermediate	14	2
I2	67	Male	5.1	*IDH2*	Yes	Intermediate	6	2
I3	68	Male	5.6	*IDH2*	Yes	Adverse	11	2

In patient N7, ∗ indicates lack of blast cell measurement in routine due to insufficient quality of the bone marrow aspirate in aplasia.

### Model design and parameter definitions

**Figure 2:**
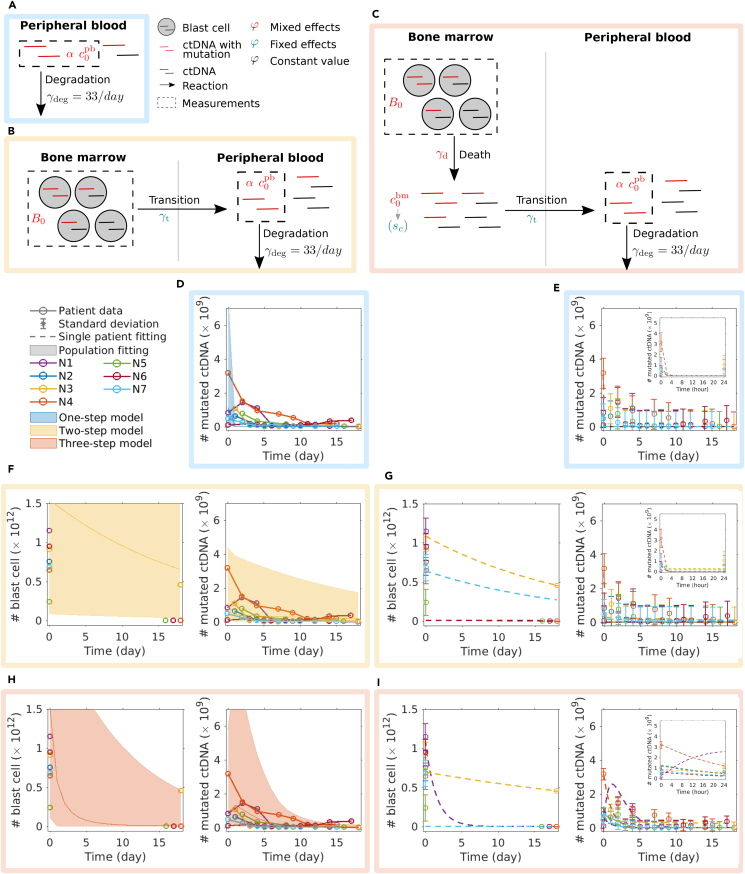
Three-step model fits population and single patient data of blast cell and ctDNA kinetics (A) A one-step model describes the kinetics of ctDNA in peripheral blood as exponential decay. (B) A two-step model considers bone marrow and peripheral blood. When blast cells die upon chemotherapeutic treatment, their ctDNA appears in peripheral blood. (C) The three-step model includes an intermediate state: After blast cells die, their ctDNA is released in the bone marrow and then transits into peripheral blood with a corresponding rate. (D) Population fitting with the one-step model (blue shade) deviates from the slow decay of the number of mutated ctDNA within days. (E) The zoom into the first 24h (inset) of the single patient fits using the one-step model reveals a rapid model decay of ctDNA within hours instead of days, as observed in the data. Data are represented as mean ± SEM. (F) Two-step model population fits estimate an overly large variance of the number of mutated ctDNAs (yellow shading). (G) Two-step model single patient fits agree well with single patient data. However, the zoom into the first 24h (inset) shows an overly steep drop in the first 4h. The standard deviation of the blast cell count is assumed to be 10% of the measured values. Data are represented as mean ± SEM. (H) Three-step model population fits agree well with blast cell numbers and the number of mutated ctDNA. (I) Three-step model single patient fits agree well with each single patient. The zoom into the first 24h (inset) shows smooth kinetics. Again, the standard deviation of the blast cell count is assumed to be 10% of the measured values. Data are represented as mean ± SEM.

The experimental data (Figure 1) revealed a heterogeneous response of AML patients during chemotherapy. To describe ctDNA and blast kinetics, we considered three models with increasing complexity (Figure 2).

• The one-step model ignores blast cells and assumes a simple exponential decay of ctDNA in peripheral blood (Figure 2A).
• The two-step model considers blast cells in the bone marrow and ctDNA in peripheral blood with a transition reaction between the two compartments (Figure 2B).
• The three-step model assumes that blast cells die and release ctDNA in the bone marrow (Figure 2C). ctDNA then transits to peripheral blood. Such an intermediate state allows for a delayed arrival of ctDNA in the peripheral blood compartment.

The ODEs of the three models and their respective parameters are listed in Tables 2 and 3. We solved all three ODE models analytically and found that the one-stage model is structurally unidentifiable, whereas the two-stage and three-stage models are structurally identifiable when both blast cell and ctDNA numbers are measured (see STAR methods for details and the corresponding derivations).

**Table 2:** Definition of three models describing ctDNA kinetics with increasing complexity

Model	One-step	Two-step	Three-step
Ordinary differential Equation	${\dot{c}}^{pb}=-\gamma_{\deg}c^{pb}$ $c^{pb}\left(0\right)=c_{0}^{pb}$ $y=\alpha c^{pb}$	$\dot{B}=-\gamma_{t}B$ ${\dot{c}}^{pb}=\gamma_{t}B-\gamma_{\deg}c^{pb}$ $B\left(0\right)=B_{0}$$c^{pb}\left(0\right)=c_{0}^{pb}$ $y=\alpha c^{pb}$	$\dot{B}=-\gamma_{d}B$${\dot{c}}^{bm}=\gamma_{d}B-\gamma_{t}c^{bm}$ ${\dot{c}}^{pb}=\gamma_{t}c^{bm}-\gamma_{\deg}c^{pb}$ $B\left(0\right)=B_{0}$$c^{bm}\left(0\right)=c_{0}^{bm}$ $c^{pb}\left(0\right)=c_{0}^{pb}$ $c_{0}^{bm}=\frac{\gamma_{\deg}}{\gamma_{t}}c_{0}^{pb}s_{c}$ $y=\alpha c^{pb}$
Fixed effects		$\gamma_{t}$	$\gamma_{t}$
Mixed effects	$c_{0}^{pb},\alpha$	$B_{0},c_{0}^{pb},\alpha$	$B_{0},c_{0}^{pb},\gamma_{d},s_{c}，\alpha$
Measurement noise	$\sigma_{c}$	$\sigma_{c}$	$\sigma_{c}$
Number of population Parameters	5	8	12
Number of patient specific Parameters	2	3	5

See STAR methods for explicit solutions.

**Table 3:** Definition of model parameters

Parameter	Description
$c_{0}^{pb}$	Total amount of all ctDNA in peripheral blood at start of treatment
$s_{c}$	Scaling factor of initial value of ctDNA in bone marrow to ensure positive gradient of $c^{pb}\left(0\right)$
$B_{0}$	Total amount of blast cells in bone marrow at the start of treatment
$\gamma_{d}$	Death rate of bone marrow blast cells during treatment in 1/days
α	Scaling factor of ctDNA in peripheral blood
$\gamma_{t}$	Transition rate of ctDNA from bone marrow to peripheral blood in 1/days. We assume this to be physiologically constrained and similar for all patients, thus handling it as a fixed effect
$\sigma_{c}$	Standard deviation of measured ctDNA

In all models, we focus on the change of the number of ctDNA molecules in a patient’s body, $dc^{pb}/dt$, and on the measurement of a subset of ctDNA molecules with a specific mutation (*NPM1* or *IDH2*), y. In the two-step and three-step models, we also consider the changing number of blast cells (independent of their mutational profile) in the bone marrow dB/dt. The number of ctDNA ($c^{pb}$) and the number of blast cells (B) is independent of the specific mutation. Only the measurement y is specific for *NPM1* and *IDH2*, respectively. Thus, the scaling factor α couples (1) the fraction of ctDNA with the specific mutation in all ctDNA and (2) the fraction of ctDNA that can be measured experimentally. Since the measurable fraction (2) should be similar for all patients, we are comparing different fractions of mutated ctDNA when we compare α between patients. A low value of α thus indicates a low fraction of specifically mutated *NPM1* or *IDH2* ctDNA and a high fraction of ctDNA molecules with other mutations.

The degradation rate of ctDNA in peripheral blood is assumed to be fixed with $\gamma_{\deg}=33/day$^25^. As the ctDNA measurement noise $\sigma_{c}$ is unknown, we estimate it simultaneously with other parameters. Furthermore, we assume 10% multiplicative measurement noise for blast cell numbers.^26^ The details of our parameter assumptions can be found in Methods.

To avoid an unphysiological drop of ctDNA in peripheral blood at the beginning of the treatment, we constrained our models. We want the derivative of $c^{pb}$ at time 0 to be non-negative:

(Equation 1) $${\dot{c}}^{pb}\left(0\right)=\gamma_{t}c_{0}^{bm}-\gamma_{\deg}c_{0}^{pb}\geq 0$$

Thus,

(Equation 2) $$c_{0}^{bm}\geq \frac{\gamma_{\deg}}{\gamma_{t}}c_{0}^{pb}$$

We can ensure this with a scaling factor *s*_*c*_ with the boundary [1,∞]:

(Equation 3) $$c_{0}^{bm}=\frac{\gamma_{\deg}}{\gamma_{t}}c_{0}^{pb}\cdot s_{c},s_{c}\geq 1$$

As *IDH2* mutated AML is only measured for three patients, we fitted *NPM1* mutated patients first.

### A three-step mixed effects model can fit both population and individual measurements

To assess the adequacy of the three proposed models for describing blast cell and ctDNA kinetics, their unknown parameters were inferred from the experimental data. Therefore, we used our internally developed MATLAB toolbox MEMOIR (https://github.com/ICB-DCM/MEMOIR) and fitted all patients simultaneously with a mixed effects modeling approach (see STAR methods for details). The parameter optimization for all three models converged and provided reproducible results (See estimated parameter values Table S2, and waterfall plots in Figure S3). The assessment of these results revealed that the one-step model does not provide a satisfactory fit due to the high reported values of the degradation rate.^25^ Both the population-level fit and the fit for individual patients is inadequately (Figures 2D and 2E). For the two-step model we observed a very large variability in the population fit (Figure 2F), but reasonable single patient fits (Figure 2G). When we use the three-step model, the population fitting agrees well with the patient data (Figure 2H), and also single patient kinetics are fitted well (Figure 2I). Comparing the single patient fits in more detail and zooming into the first 24 h, we see a very steep decrease when using the two-step model (Figure 2G, inset). In contrast, the three-step model shows a short increase of ctDNA in the peripheral blood before levels drop. Based on these model fits, we continue analyzing the three-step model.

### Blast cell numbers can be predicted given estimated parameters and ctDNA data

**Figure 3:**
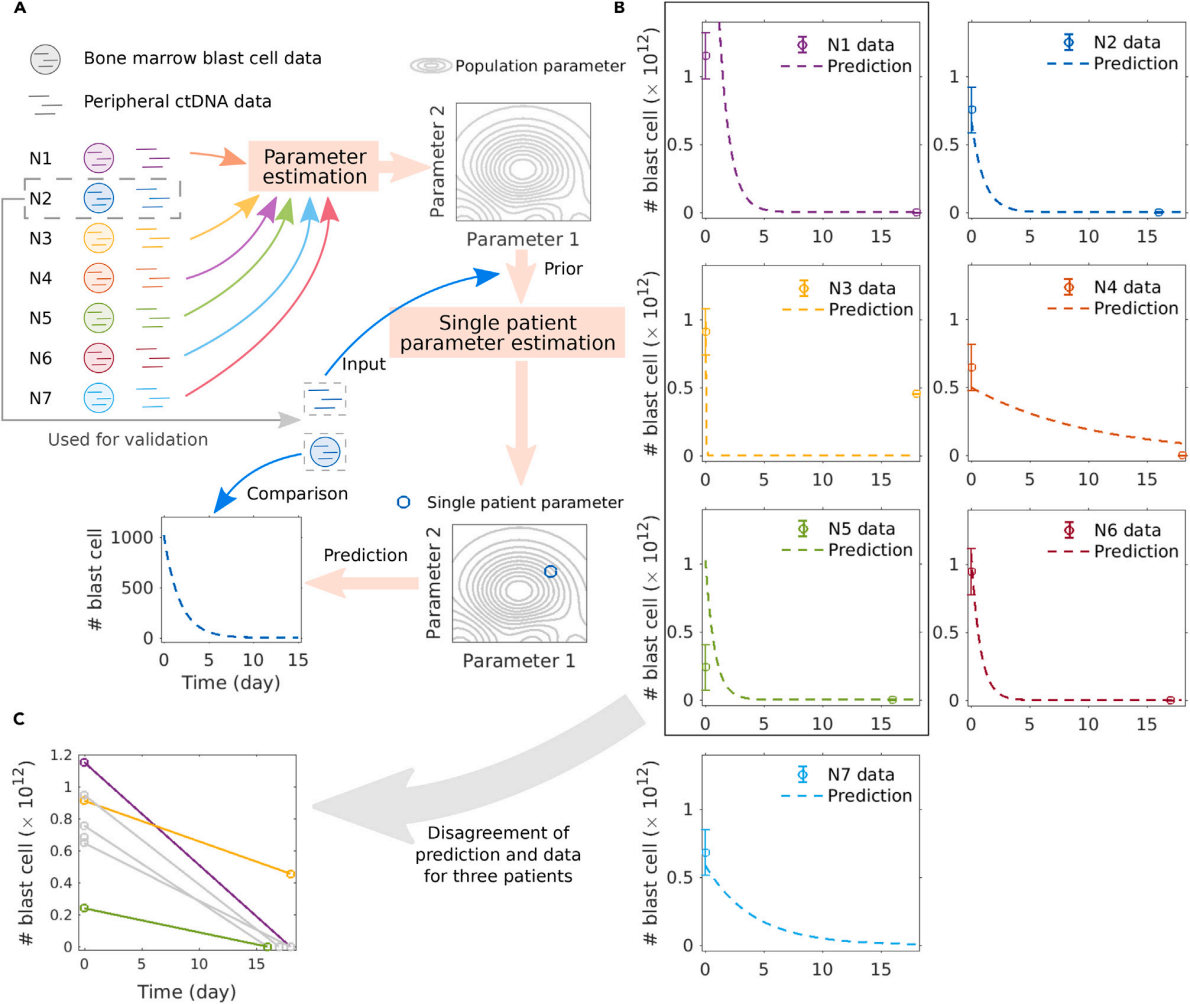
Three-step model predicts the kinetics and scale of bone marrow blast cells during the first days of chemotherapy from peripheral blood ctDNA measurements (A) Predicting bone marrow blast cell numbers using only ctDNA data. One patient (N2 in this exemplary case) is excluded when estimating the population parameters of the three-step model. Next, the ctDNA data of N2 is used as input, combined with the population parameters as prior, and the single patient parameters of N2 are estimated. For validation, the predicted blast cell number is compared with the true patient data. (B) Comparison of predicted bone marrow blast cell number and patient data. Bone marrow data of four out of seven patients can be predicted accurately. Data are represented as mean ± SEM. (C) N1 (violet) and N5 (green) constitute the upper and lower boundaries of the measured ctDNA numbers. The high blast counts at the second measurement for N3 (yellow) are due to a primary refractory disease.

To assess the value of the three-step model, we test the prediction performance for the absolute bone marrow blast cell numbers from ctDNA measurements in peripheral blood. We assume that we have only measured ctDNA from a patient, and combine this as a prior with the population parameters estimated from the other patients (Figure 3A). With that approach, the absolute bone marrow blast cell numbers can be predicted and compared with the measured data. We validated our method by excluding each patient separately (Figure 3A).

Results show that we hit the 10% variance of the measured blast cell numbers for four out of seven patients (Figure 3B). The disagreement of the prediction for patients N1 and N4 (Figure 3B) can be explained by the fact that the blast cell measurement of those two patients are upper and lower limits in our dataset (Figure 3C). We also fail to predict the high second blast cell measurement of patient N3, 18 days after initiation of therapy, due to persistence of blasts after induction chemotherapy.

### Kinetics of patients with *NPM1* and *IDH2* mutations can be fit using one set of parameters

The ctDNA measurements of *NPM1* and *IDH2* mutated AML patients are on the same scale (Figure 1). Therefore, we tried to fit the data from the two patient groups simultaneously.

**Figure 4:**
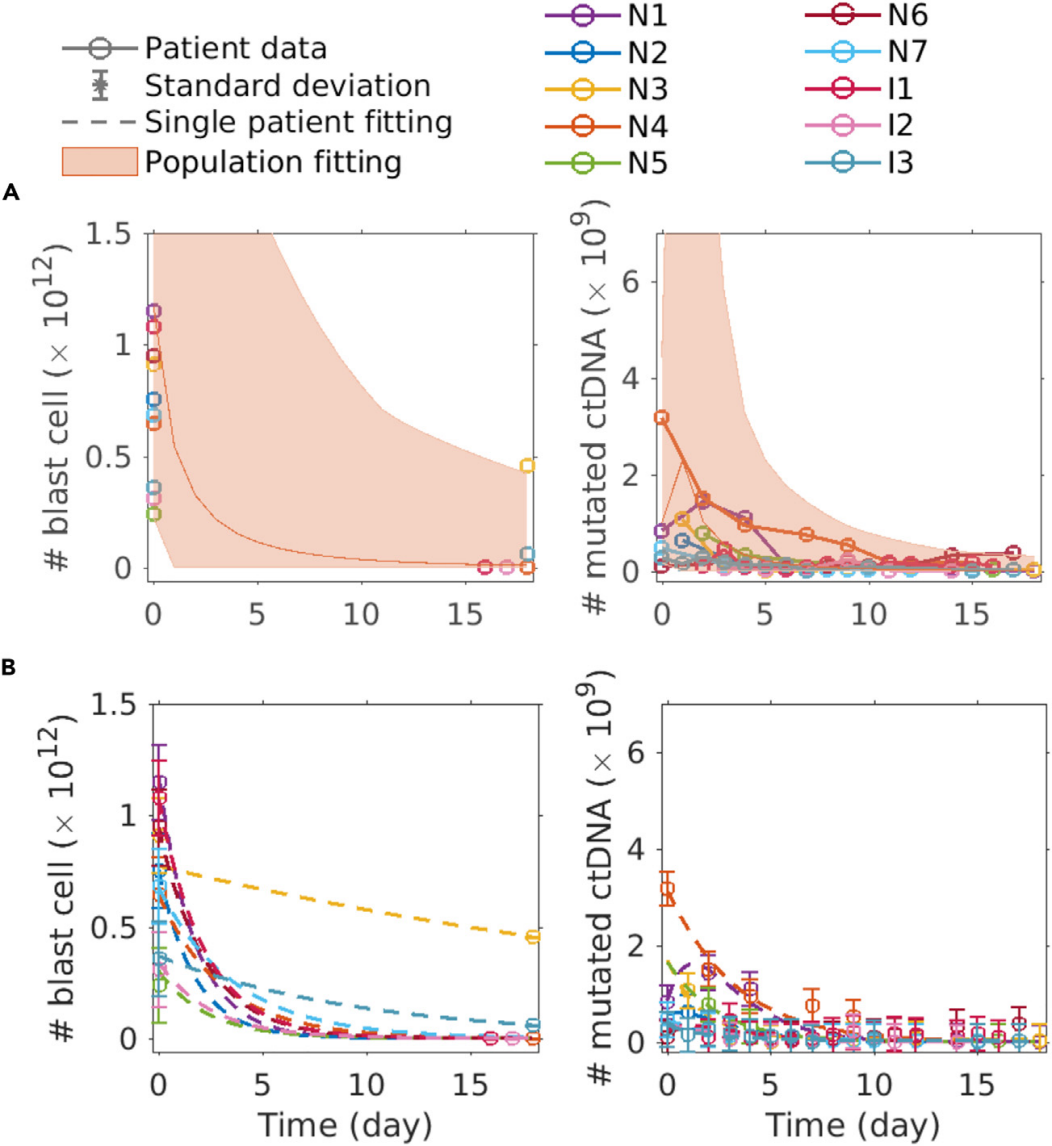
The three-step model is able to fit *NPM1* and *IDH2* mutated patients simultaneously (A) Population fitting. (B) Single patient fitting. The second blast cell measurement of patient N3 with different kinetics can also be fitted, meaning that our model is flexible enough to fit the different kinetics. Data are represented as mean ± SEM.

Results show that data from both mutations can be fit simultaneously, and that the population and single patient fitting agree well with patient data (Figure 4). Compared to the population fittings using only *NPM1* mutated data, the variance of predicted ctDNA data are larger though.

### Single patient parameters indicate disease relapse

**Figure 5:**
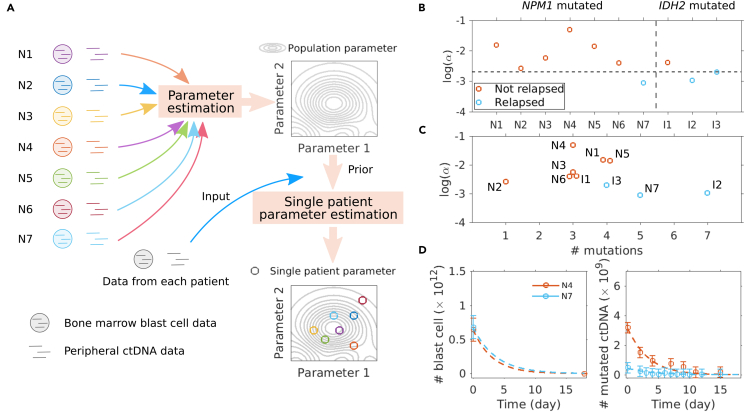
Small fraction of measured mutations correlates with relapse (A) Pipeline of computing single patient parameters. All patient data are used to estimate the population parameters, which are then given as a prior, and all single patient data are used to estimate the corresponding parameters. (B) Values of measurement scaling factor α for each single patient. (C) Values of α versus number of mutations. (D) Single patient fitting of patient N4 and N7 with similar blast cell kinetic and different ctDNA measurements. Data are represented as mean ± SEM.

To investigate the relationship between inferred parameter values and disease relapse, we compute single patient parameters using the population parameter as a prior (Figure 5A). By comparing all parameters (Figure S1), we found for the three relapsed patients (blue circles in Figure 5B) the scaling factor α to be smaller than for the seven non-relapsed patients. This holds when using only *NPM1* mutated data, or both mutations for inferring single patient parameters. Note that by giving the population parameters as a prior, the single patient parameter α is identifiable (Figure S2).

As α is the fraction of ctDNA with the specific mutation in the total amount of ctDNA, small α indicates that also fractions of ctDNA from other blast cell clones with mutations that we have not measured are present, i.e., this could reflect a larger heterogeneity of AML related mutations (Figure 5C). Comparing patients N4 and N7 (Figure 5D) who show very similar bone marrow blast cell numbers and kinetics, we see that the ctDNA level of patient N7 is much lower than for patient N4. As we specifically measured the *NPM1* mutation within the ctDNA, the small measured fraction means that for patient N7, also a fraction of ctDNA containing other AML related mutations has to be present. Indeed patient N7 had 5 different mutations of clinical significance and one variant of unknown significance detected by a routine gene panel, while in patient N4 only 4 mutations of clinical significance could be identified (Figure 5C; Table S1). This agrees with the fact that patients with a more heterogeneous clone composition have a dismal overall prognosis.^27^ Therefore, when only measurements of one specific mutation are available, one can still predict the prognosis by computing the α value using the three-step model.

## Discussion

Recent advances in AML have led to improved risk stratification and approval of new therapies to achieve complete remission.^21^^,^^28^ The best long-term outcome for intensively treated patients depends largely on early clearance of leukemic blasts in the bone marrow and negativity of measurable residual disease (MRD), which may have stronger prognostic significance than pretreatment genetic risk.^29^^,^^30^^,^^31^ For assessing treatment response, bone marrow biopsy remains the gold standard, as the sensitivity of MRD analyses from bone marrow is at least one log-fold higher than the same analysis from peripheral blood).^20^^,^^32^ Thus, 16–21 days after intensive induction chemotherapy, the percentage of remaining blasts in the bone marrow is determined. This timing is critical to guide further treatment, such as a second intensive induction chemotherapy. However, determining blast counts in bone marrow is a burden for the patient, and can be difficult in aplastic bone marrow due to aspicular samples, dilution with peripheral blood, or changes associated with early marrow recovery.^33^^,^^29^ Sometimes marrow analysis must be repeated after leukocyte regeneration. To address these challenges, we developed a mathematical model to predict blast cells in the bone marrow at this early time point by analyzing ctDNA from peripheral blood of AML patients. In 4 of 7 patients, our predictions are within 10% of the measured data. Our finding that two cases at the parameter limits cannot be predicted correctly gives us confidence that bone marrow prediction will work for the majority of patients in larger cohorts.

In contrast to ctDNA, other liquid biopsies components such as cell free DNA (cfDNA) or circulating tumor cells (CTCs) seem to be less useful for therapy monitoring in AML. cfDNA is shedded into the bloodstream by all cells and most DNA fragments do not harbor tumor specific mutations.^34^ Peaks in cfDNA amount have been shown to also occur due to increased cell turnover during infection or regeneration.^20^^,^^35^ Therefore the analysis of ctDNA as a tumor specific fraction of cfDNA seems beneficial for monitoring cancer. While CTCs are widely studied in solid cancers, their application in AML seems not feasible, as detection of CTCs usually depends on the larger size of cancer cells or specific surface markers.^36^ Leukemic blasts do not differ in these characteristics from benign stem and progenitor cells making it infeasible to specifically capture leukemic CTCs.

The potential of ctDNA to assess treatment response has been shown in a wide variety of solid tumors^37^ and is a feasible tool to follow-up hematologic cancers, such as lymphoma and leukemia.^20^^,^^38^^,^^39^^,^^40^ Combined with mathematical modeling, it outcompetes the standard, computed tomography imaging based follow-up measurements in solid cancers.^41^ Using the percentage of bone marrow blast cells in combination with mutation-specific ctDNA measurements in peripheral blood, we estimated population parameters of a mixed effect model for a cohort of 7 *NPM1* and 3 *IDH2* mutated AML patients. The model was selected in a stepwise way, beginning with a simple one-step model to prevent overfitting,^42^ and increasing complexity. Our three-step model, where leukemic cells die within the bone marrow and release ctDNA that transits into the bloodstream where it degrades with a fixed rate, was able to fit the clinical data. One parameter of this model, a patient-specific scaling factor α, is small for relapsing patients, independent of the ELN risk group.^21^ Since α quantifies the ratio of ctDNA with a specific mutation to the amount of total ctDNA, we assume that this parameter is informative about the tumor heterogeneity and might be suitable to identify patients who are at risk for relapse. However, with our small cohort and only one mutation measured in ctDNA per patient we cannot conclude on the clonal trajectory of AML, in particular as the clonal architecture of AML can be quite complex^43^: Even mutations considered early in leukemogenesis, such as *NMP1*, may be preceded by other mutations and thus may not be present in all AML clones. While α is not informative about the mutations present at relapse, we found only minor changes in the mutational spectrum of our relapsed patients. Of note, the mutations we tracked (*NPM1* or *IDH2*) were still present at relapse because, as previously shown, *NPM1*-negative relapse in AML is quite rare when *NPM1* is present at initial diagnosis.^44^ Because the presence of ctDNA is largely dependent on release from dying tumor cells and passage of ctDNA into the circulation, follow-up studies are needed to further investigate whether the clonal architecture of AML can be detected in ctDNA and whether different AML clones that respond differently to therapy also exhibit different ctDNA kinetics.

Interestingly, we are able to calculate the scaling factor α already when only 2 measurements of the ctDNA at early and late time points are performed. This excludes that the calculation of α is influenced by other sources of cell-free DNA (e.g., regeneration of healthy hematopoiesis after induction therapy). Thus our model might be used to refine risk assessment in AML.

However, because only a limited number of clinical measurements at different time points and a total of only 10 patients were available, we had to restrict the model parameters to improve their identifiability. Therefore, we fixed the degradation rate for ctDNA to the value previously estimated by monitoring fetal cell-free DNA in women after delivery.^25^ Although degradation of ctDNA and cell-free DNA should not differ, we cannot exclude cancer-specific effects due to inflammation or chemotherapy. Identifying ctDNA degradation rates in cancer patients would help validate our model for AML and previous models for solid cancers. Remarkably, all model parameters are identifiable. Another assumption of the model is that the number of bone marrow cells is constant. Although this number is certainly capped by physical constraints, it could be higher at the start of chemotherapy when blasts are abundant than at the second measurement over 2 weeks later. However, because blast measurements at this time are 0 in 8 of 10 patients, we believe that errors from this assumption affecting the parameter distributions of the two patients with nonzero measurements (patients N3 and I3) are negligible.

A larger cohort of patients could give us insight into the degree of variability between AML patients, especially with different mutation patterns, as we have only used ctDNA from two genes commonly mutated in AML, *NPM1* and *IDH2*. To analyze more than one mutation within the same patient over time a next-generation sequencing (NGS) panel from liquid biopsies would be required for AML, comparable to those commercially available for solid tumors and already used in precision oncology programs when tumor biopsies cannot be obtained.^45^ Such a panel would also allow us to more reliably assess the impact of scaling factor α. It also has the potential to add relevant information to existing NGS panels from bone marrow aspirates and liquid biopsies, as it contains mutations that could only be identified using a whole genome sequencing approach.^46^^,^^47^ A model based on parameters estimated from such a larger and more diverse group of patients could then be used in a multicenter cohort to predict treatment response and relapse risk. Because recent studies highlight the importance of MRD monitoring in AML patients regardless of treatment and the high risk of relapse for patients who do not achieve MRD negativity,^30^ we believe that our model could also guide MRD monitoring by identifying relevant mutations that do not decrease during induction chemotherapy and should be followed in AML patients for MRD assessment.

In summary, we developed a mechanistic model to describe early response to intensive induction chemotherapy in AML patients. We applied it to clinical data and showed that it is able to predict blast cell clearance from ctDNA data and might be able to predict relapse risk also in patients with favorable or intermediate disease. Using ctDNA measurements and disease modeling of AML might help to gain deeper insight into therapy response and clonal heterogeneity of AML. Our modeling approach might guide measurable disease monitoring and enhance risk stratification to identify patients at high risk for initial refractory disease or to develop relapses.

### Limitations of the study

Our model identified the scaling factor to predict relapse risk in AML and is able to model therapy response during initial intensive chemotherapy. So far we have only studied ctDNA kinetics in a small cohort of patients and further confirmation in a larger cohort will be necessary. Moreover, we assessed ctDNA kinetics only in two mutations within AML patients. Measuring ctDNA kinetics of other frequently mutated genes in AML might help to improve our model. Finally, we had to fix some parameters in the model, due to the small cohort size. We are aware that some factors might be more heterogeneous and differ from patient to patient. A larger and more diverse cohort would enable us to overcome these limits and improve fitting and predictive power of the model.

## STAR★Methods

### Key resources table

**Table:** 

REAGENT or RESOURCE	SOURCE	IDENTIFIER
**Deposited data**		

NPM1 and IDH2 mutated data	Christian, Maja Rothenberg-Thurley, Simon A. Buerger, Sebastian Tschuri, Annika Dufour, Michaela Neusser, Stephanie Schneider, Karsten Spiekermann, Klaus H. Metzeler, and Frank Ziemann. 2021. “Double Drop-Off Droplet Digital PCR: A Novel, Versatile Tool for Mutation Screening and Residual Disease Monitoring in Acute Myeloid Leukemia Using Cellular or Cell-Free DNA.” *The Journal of Molecular Diagnostics: JMD* 23 (8): 975–85	https://doi.org/10.1016/j.jmoldx.2021.05.001

**Software and algorithms**		

MATLAB R2017a	Mathworks	https://www.mathworks.com/
PESTO	Stapor, Paul, Daniel Weindl, Benjamin Ballnus, Sabine Hug, Carolin Loos, Anna Fiedler, Sabrina Krause, Sabrina Hroβ, Fabian Fröhlich, and Jan Hasenauer. 2018. “PESTO: Parameter EStimation TOolbox.” *Bioinformatics* 34 (4): 705–7.	https://github.com/ICB-DCM/PESTO
AMICI	Fröhlich F, Weindl D, Schälte Y et al. AMICI: high-performance sensitivity analysis for large ordinary differential equation models[J]. Bioinformatics, 2021, 37(20): 3676–3677.	https://github.com/AMICI-dev/AMICI
SPToolbox		https://github.com/ICB-DCM/SPToolbox
MEMOIR		https://github.com/ICB-DCM/MEMOIR
code-model simulation	this paper	https://doi.org/10.5281/zenodo.8361859

### Resource availability

#### Lead contact

Further information and requests should be directed to and will be fulfilled by the Lead Contact Carsten Marr (carsten.marr@helmholtz-munich.de).

#### Materials availability

This study did not generate new materials.

#### Data and code availability

• The pre-processed patients data can be found in zenodo, link can be found in the key resources table.
• We implemented the log likelihood of patient time-lapse data in the MATLAB toolbox MEMOIR (https://github.com/ICB-DCM/MEMOIR) with the MATLAB version of R2017b. To solve the optimization problem, we employed multi-start local optimization via the open-source MATLAB toolbox PESTO (https://github.com/ICB-DCM/PESTO).^48^ The local optimization was performed using the MATLAB function **fmincon** with interior-point method. Code of ODE model and parameter simulation in this manuscript can be found in zenodo, link is listed in the key resources table.

### Experimental model and subject details

Peripheral blood samples from patients with AML treated at the LMU University Hospital were collected during induction therapy and further processed for ctDNA analysis, as previously described.^20^ Samples were further processed to isolate ctDNA according to manufacturer’s instructions. ddPCR was performed with self designed double drop–off assays, as previously described.^20^ Isolated ctDNA was independently quantified using the Agilent 2100 Bioanalyzer with the Agilent High Sensitivity DNA Kit (Agilent, Santa Clara, CA, USA) twice and analyzed at least two times with viaddPCR.

#### Ethics

All data for this study was collected in the Department of Hematology and Oncology at the LMU hospital between February 2018 and August 2020. The sampling of patient material and ctDNA analysis was approved by the local ethics committee in accordance with the Declaration of Helsinki (approval number 18–539). All patients provided written informed consent.

#### Ethics approval

Our study was approved by the local ethics committee in accordance with the Declaration of Helsinki (approval number 18–539). All patients provided written informed consent.

### Method details

#### Mixed effects modeling

To take patient to patient variability into account, we used a nonlinear mixed effects model. The time evolution of the state variables x, e.g., the amount of ctDNA in peripheral blood or blast cells in bone marrow, was described with ODEs:

$$\dot{x}=f\left(x,\exp\left(\varphi \right)\right)$$

We use the term ‘mixed effects’ for φ to distinguish it from parameters that need to be estimated later. In line with their biological definition, all effects are exponentially transformed to insure positivity. The mixed effects are defined as a linear combination of fixed effects β and random effects b,

φ=Aβ+Bb,b∼N(0,D)

with design matrices A and B, and covariance matrix D. We assume that there is no correlation between the random effects, so

D=exp(diag(δ)).

Here, δ is the vector of variances.

Often, experimental techniques do not provide direct measurements, but only a subset or transformations of x. In our case, the percentage of blast cells in bone marrow and ctDNA concentrations in peripheral blood are measured instead of numbers. Therefore, we define outputs

y(t,φ)=h(x(t,φ),φ)

using the function h() mapping x to observables y.

To handle the time-lapse nature of the data, we implemented a first-order conditional approximation method and added it to the MEMOIR toolbox (https://github.com/ICB-DCM/MEMOIR). Parameters were estimated using a multi-start local optimization approach with 300 start points. The local optimizations were performed using the trust-region method fmincon, with gradients computed using forward sensitivity analysis.

#### Data preprocessing

To decrease the number of parameters that need to be estimated, we constrained parameters using information found in the literature.

1. Our measured data in the bone marrow compartment is the percentage of blast cells in all nucleated cells, $p^{bm}$. To compute the total number of blast cells, B, we assume that the nucleated cell number in the bone marrow is $1.2\cdot 10^{12}$^24^: m$B=1.2\cdot 10^{12}p^{b}$.
2. ctDNA is measured as concentration (see STAR methods) $C^{pb}\left(1/L\right)$. To compute the total amount of ctDNA $c^{pb}$ in peripheral blood, we calculated the individual blood volume V for each patient using body weight and height as described in^23^: b$c^{pb}=VC^{p}$.
3. The first measurement is typically taken 1–6 days before the chemotherapy treatment. Similar to Almendero et al.,^49^ we assume that the blast cells do not proliferate dramatically in between and therefore we use the measured data as the initial point of the modeled period.

#### Parameter assumptions

• As we do not have multiple measurements to assess measurement noise, we assume a multiplicative noise of 10%.^26^ For the 0 measurement values (at day 16), we manually set the noise to the noise of the patients with non-zero measurement values at that time point.
• We assume the degradation rate of ctDNA to be $\gamma_{\deg}=33/\text{day}$, which equals a half-life of 30 min.^25^.
• The bone marrow of AML patients is usually completely packed with leukemic blasts. We thus assume that every blast is carrying an AML mutation, and that the amount of healthy, unmutated bone marrow cells is neglectable.

#### Analytical solution and model identifiability

By solving all three models in Table 1 analytically, we get the solution as following,

• One-step model:

$$c^{pb}\left(t\right)=c_{0}^{pb}\;\exp\left(-\gamma_{\deg}t\right)$$

$$y=\alpha c^{pb}$$

• Two-step model:

$$B\left(t)=B_{0}\;\exp(-\gamma_{t}t\right)$$

$$if\;\gamma_{\deg}=\gamma_{t}=\gamma \text{.}$$

$$c^{pb}(t)=\gamma B_{0}t\;exp(-\gamma t)+c_{0}^{pb}\;exp(-\gamma t)$$

$$\text{if}\;\gamma_{\deg}\neq \gamma_{t}\text{,}$$

$$c^{pb}\left(t\right)=\frac{\gamma_{t}B_{0}}{\gamma_{\deg}-\gamma_{t}}\exp\left(-\gamma_{t}t\right)+\left(c_{0}^{pb}-\frac{\gamma_{t}B_{0}}{\gamma_{\deg}-\gamma_{t}}\right)\exp\left(-\gamma_{\deg}t\right)$$

$$y_{1}=\alpha c^{pb}$$

$$y_{2}=B$$

• Three-step model

$$B\left(t)=B_{0}\;\exp(-\gamma_{d}t\right)$$

$$if\;\gamma_{t}=\gamma_{d}=\gamma \text{,}$$

$$c^{bm}(t)=\gamma B_{0}t\;exp(-\gamma t)+C_{0}^{bm}\;exp(-\gamma t)$$

$$if\;\gamma_{t}\neq \gamma_{d}\text{,}$$

$$c^{bm}(t)=\frac{\gamma_{d}B_{0}}{\gamma_{t}-\gamma_{d}}exp(-\gamma_{d}t)+\left(c_{0}^{bm}-\frac{\gamma_{d}B_{0}}{\gamma_{t}-\gamma_{d}}\right)exp(-\gamma_{t}t)$$

$$if\;\gamma_{\deg}=\gamma_{t}=\gamma_{d}=\gamma \text{,}$$

$$c^{pb}(t)=(\frac{1}{2}\gamma^{2}B_{0}t^{2}+c_{0}^{pb})\exp\left(-\gamma t\right)$$

$$if\;\gamma_{\deg}\neq \gamma_{t},\gamma_{t}=\gamma_{d}=\gamma \text{,}$$

$$c^{pb}\left(t\right)=\left(\frac{\gamma^{2}B_{0}}{\gamma_{\deg}-\gamma }t-\frac{\gamma^{2}B_{0}}{{\left(\gamma_{\deg}-\gamma \right)}^{2}}+\frac{\gamma c_{0}^{bm}}{\gamma_{\deg}-\gamma }\right)\exp\left(-\gamma t\right)+\left(c_{0}^{pb}-\frac{\gamma^{2}B_{0}}{{\left(\gamma_{\deg}-\gamma \right)}^{2}}-\frac{\gamma c_{0}^{bm}}{\gamma_{\deg}-\gamma }\right)\exp\left(-\gamma_{\deg}t\right)$$

$$if\;\gamma_{\deg}=\gamma_{t}=\gamma_{1},\gamma_{t}\neq \gamma_{d}$$

$$c^{pb}\left(t\right)=\frac{\gamma_{1}\gamma_{d}B_{0}}{{\left(\gamma_{1}-\gamma_{d}\right)}^{2}}\exp(\gamma_{d}t)+\left(c_{0}^{bm}-\frac{\gamma_{d}B_{0}}{\gamma_{1}-\gamma_{d}}\right)t\;exp(-\gamma_{1}t)+\left(c_{0}^{pb}-\frac{\gamma_{1}\gamma_{d}B_{0}}{{\left(\gamma_{1}-\gamma_{d}\right)}^{2}}\right)exp(-\gamma_{1}t)$$

$$if\;\gamma_{\deg}=\gamma_{d}=\gamma_{2},\gamma_{d}\neq \gamma_{t}\text{,}$$

$$c^{pb}(t)=\frac{\gamma_{t}\gamma_{2}B_{0}}{\gamma_{t}-\gamma_{2}}t\;exp(-\gamma_{2}t)+\left(\frac{c_{0}^{bm}}{\gamma_{2}-\gamma_{t}}+\frac{\gamma_{2}B_{0}}{{\left(\gamma_{2}-\gamma_{t}\right)}^{2}}\right)exp(-\gamma_{t}t)+\left(c_{0}^{pb}-\frac{c_{0}^{bm}}{\gamma_{2}-\gamma_{t}}-\frac{\gamma_{2}B_{0}}{{\left(\gamma_{2}-\gamma_{t}\right)}^{2}}\right)exp(-\gamma_{2}t)$$

$$if\;\gamma_{\deg}\neq \gamma_{t}\neq \gamma_{d}\text{,}$$

$$c^{pb}(t)=\frac{\gamma_{t}\gamma_{d}B_{0}}{\left(\gamma_{deg}-\gamma_{d}\right)\left(\gamma_{t}-\gamma_{d}\right)}exp(-\gamma_{d}t)+\frac{\left(\gamma_{t}-\gamma_{d}\right)\gamma_{t}c_{0}^{bm}-\gamma_{t}\gamma_{d}B_{0}}{\left(\gamma_{deg}-\gamma_{t}\right)\left(\gamma_{t}-\gamma_{d}\right)}exp(-\gamma_{t}t)$$

$$+\left(c_{0}^{pb}+\frac{\gamma_{t}\gamma_{d}B_{0}}{\left(\gamma_{t}-\gamma_{d}\right)\left(\gamma_{deg}-\gamma_{d}\right)}-\frac{\gamma_{t}c_{0}^{bm}}{\gamma_{deg}-\gamma_{t}}+\frac{\gamma_{t}\gamma_{d}B_{0}}{\left(\gamma_{deg}-\gamma_{t}\right)\left(\gamma_{t}-\gamma_{d}\right)}\right)exp\left(-\gamma_{deg}t\right)$$

$$y_{1}=\alpha c^{pb}$$

$$y_{2}=B$$

It can be seen that in the one-step model the parameters $c_{0}^{pb}$ and α have the same effects on the output y. Therefore, the one-step model parameters are non-identifiable. We have also checked the identifiability of the models using the MATLAB toolbox GenSSI 2.0.^50^ We see that if both blast cell number and ctDNA concentration are provided as measurement, the two-step model and three-step model are structurally identifiable. If only ctDNA concentration is provided, then one of the parameters is not structurally identifiable.

#### Derivatives of outputs with respect to parameters

Here we show the derivatives of the outputs of all three models with respect to the model parameters.

• One-step model

$$\frac{dy}{dc_{0}^{pb}}=\alpha \;\exp\left(-\gamma_{\deg}t\right)$$

$$\frac{dy}{d\alpha }=c_{0}^{pb}\;\exp\left(-\gamma_{\deg}t\right)$$

$$\frac{dy}{d\gamma_{\deg}}=\alpha c_{0}^{pb}t\;\exp\left(-\gamma_{\deg}t\right)$$

• Two-step model

$$\frac{dy_{2}}{dB_{0}}=\exp\left(-\gamma_{t}t\right)$$

$$\frac{dy_{2}}{d\gamma_{t}}=-B_{0}t\;\exp\left(-\gamma_{t}t\right)$$

$$if\;\gamma_{\deg}=\gamma_{t}=\gamma \text{,}$$

$$\frac{dy_{1}}{dB_{0}}=\alpha \gamma_{t}t\;\exp\left(-\gamma_{\deg}t\right)$$

$$\frac{dy_{1}}{dc_{0}^{pb}}=\alpha \;\exp\left(-\gamma_{\deg}t\right)$$

$$\frac{dy_{1}}{d\alpha }=\gamma B_{0}t\;\exp\;t\left(-\gamma t\right)+c_{0}^{pb}\;\exp\left(-\gamma t\right)$$

$$\frac{dy_{1}}{d\gamma_{t}}=\alpha B_{0}t\;\exp\left(-\gamma_{\deg}t\right)$$

$$\frac{dy_{1}}{d\gamma_{\deg}}=\alpha (-\gamma_{t}B_{0}t^{2}\;\exp(-\gamma_{\deg}t)-c_{0}^{pb}t\;\exp(-\gamma_{\deg}t))$$

$$if\;\gamma_{\deg}\neq \gamma_{t}\text{,}$$

$$\frac{dy_{1}}{dB_{0}}=\alpha \left(\frac{\gamma_{t}}{\gamma_{\deg}-\gamma_{t}}\exp\left(-\gamma_{t}t\right)-\frac{\gamma_{t}}{\gamma_{\deg}-\gamma_{t}}\exp\left(-\gamma_{\deg}t\right)\right)$$

$$\frac{dy_{1}}{dc_{0}^{pb}}=\alpha \;\exp\left(\gamma_{\deg}t\right)$$

$$\frac{dy_{1}}{d\alpha }=\frac{\gamma_{t}B_{0}}{\gamma_{\deg}-\gamma_{t}}\exp\left(-\gamma_{t}t\right)+\left(c_{0}^{pb}-\frac{\gamma_{t}B_{0}}{\gamma_{\deg}-\gamma_{t}}\right)\exp\left(-\gamma_{\deg}t\right)$$

$$\frac{dy_{1}}{d\gamma_{t}}=\alpha \left(-\frac{\gamma_{t}B_{0}}{\gamma_{\deg}-\gamma_{t}}t\;\exp\left(-\gamma_{t}t\right)+\frac{\gamma_{\deg}}{{\left(\gamma_{\deg}-\gamma_{t}\right)}^{2}}\exp\left(-\gamma_{t}t\right)-\frac{\gamma_{\deg}}{{\left(\gamma_{\deg}-\gamma_{t}\right)}^{2}}\exp\left(-\gamma_{\deg}t\right)\right)$$

$$\frac{dy_{1}}{d\gamma_{\deg}}=\alpha \left(\frac{\gamma_{t}}{{\left(\gamma_{\deg}-\gamma_{t}\right)}^{2}}\exp\left(-\gamma_{t}t\right)+\frac{\gamma_{t}}{\gamma_{\deg}-\gamma_{t}}t\;\exp\left(-\gamma_{\deg}t\right)-\frac{\gamma_{t}}{{\left(\gamma_{\deg}-\gamma_{t}\right)}^{2}}\exp\left(-\gamma_{\deg}t\right)\right)$$

• Three-step model

$$\frac{dy_{2}}{dB_{0}}=\exp\left(-\gamma_{d}t\right)$$

$$\frac{dy_{2}}{d\gamma_{t}}=-B_{0}t\;\exp\left(-\gamma_{d}t\right)$$

$$if\gamma_{\deg}=\gamma_{t}=\gamma_{d}=\gamma \text{,}$$

$$\frac{dy_{1}}{dB_{0}}=\frac{1}{2}\alpha \gamma^{2}t^{2}\;\exp\left(-\gamma t\right)$$

$$\frac{dy_{1}}{dc_{0}^{pb}}=\alpha \;\exp\left(-\gamma t\right)$$

$$\frac{dy_{1}}{d\alpha }=\left(\frac{1}{2}\gamma^{2}B_{0}t^{2}+\gamma c_{0}^{bm}\right)t\;\exp\left(-\gamma t\right)+\left(c_{0}^{pb}-\gamma c_{0}^{bm}\right)\exp\left(-\gamma t\right)$$

$$\frac{dy_{1}}{d\gamma_{t}}=\alpha \left(\frac{1}{2}\gamma B_{0}t^{2}+c_{0}^{bm}t-\frac{1}{3}\gamma^{2}B_{0}t^{3}-\frac{1}{2}c_{0}^{bm}t^{3}\right)\exp\left(-\gamma t\right)$$

$$\frac{dy_{1}}{d\gamma_{d}}=\alpha \left(\frac{1}{2}\gamma B_{t}t^{2}-\frac{1}{6}\gamma^{2}B_{0}t^{3}\right)\exp\left(-\gamma t\right)$$

$$\frac{dy_{1}}{d\gamma_{\mathrm{de}g}}=\alpha \left(-\frac{1}{6}\gamma^{2}B_{0}t^{3}+\frac{3}{2}\gamma c_{0}^{bm}t^{2}+c_{0}^{pb}t+\gamma c_{0}^{bm}t\right)\exp\left(-\gamma t\right)$$

$$if\;\gamma_{\deg}\neq \gamma_{t},\gamma_{t}=\gamma_{d}=\gamma \text{,}$$

$$\frac{dy_{1}}{dB_{0}}=\alpha \left(\left(\frac{\gamma^{2}}{\gamma_{\deg}-\gamma }t-\frac{\gamma^{2}}{{\left(\gamma_{\deg}-\gamma \right)}^{2}}\right)\exp\left(-\gamma t\right)+\left(\frac{\gamma^{2}}{{\left(\gamma_{\deg}-\gamma \right)}^{2}}\right)\exp\left(-\gamma_{\deg}t\right)\right)$$

$$\frac{dy_{1}}{dc_{0}^{pb}}=\alpha \;\exp\left(-\gamma_{\deg}t\right)$$

$$\frac{dy_{y}}{dc_{0}^{bm}}=\alpha \left(\frac{\gamma }{\gamma_{\deg}-\gamma }\exp\left(-\gamma t\right)-\frac{\gamma }{\gamma_{\deg}-\gamma }\exp\left(-\gamma_{\deg}t\right)\right)$$

$$\frac{dy_{1}}{d\alpha }=\left(\frac{\gamma^{2}B_{0}}{\gamma_{\deg}-\gamma }t-\frac{\gamma^{2}B_{0}}{{\left(\gamma_{\deg}-\gamma \right)}^{2}}+\frac{\gamma c_{0}^{bm}}{\gamma_{\deg}-\gamma }\right)\exp\left(-\gamma t\right)+\left(c_{0}^{pb}-\frac{\gamma^{2}B_{0}}{{\left(\gamma_{\deg}-\gamma \right)}^{2}}-\frac{\gamma c_{0}^{bm}}{\gamma_{\deg}-\gamma }\right)\exp\left(-\gamma_{\deg}t\right)$$

$$\frac{dy_{1}}{d\gamma_{t}}=\alpha \left(\frac{2}{{\left(\gamma_{\deg}-\gamma \right)}^{3}}+\frac{\gamma \gamma_{\deg}B_{0}t-1}{{\left(\gamma_{\deg}-\gamma \right)}^{2}}-\frac{\gamma c_{0}^{bm}t-0.5\gamma^{2}B_{0}t^{2}+c_{0}^{bm}}{\gamma_{\deg}-\gamma }\right)\exp\left(-\gamma t\right)$$

$$\frac{dy_{1}}{d\gamma_{d}}=\alpha \left(\frac{\gamma \gamma_{\deg}B_{0}t-1}{{\left(\gamma_{\deg}-\gamma \right)}^{2}}-\frac{0.5\gamma^{2}B_{0}t^{2}}{\gamma_{\deg}-\gamma }-\frac{1}{{\left(\gamma_{\deg}-\gamma \right)}^{3}}\right)\exp\left(-\gamma t\right)$$

$$\frac{dy_{1}}{d\gamma_{\deg}}=\alpha \left(-\frac{1}{6}\gamma^{2}B_{0}t^{3}+\frac{3}{2}\gamma c_{0}^{bm}t^{2}+\gamma c_{0}^{bm}t+c_{0}^{pb}t\right)\exp\left(-\gamma_{\deg}t\right)$$

$$if\;\gamma_{\deg}=\gamma_{t}=\gamma_{1},\gamma_{t}\neq \gamma_{d}\text{,}$$

$$\frac{dy_{1}}{dB_{0}}=\alpha \left(\frac{\gamma_{1}\gamma_{d}}{{\left(\gamma_{1}-\gamma_{d}\right)}^{2}}\exp\left(\gamma_{d}t\right)+\frac{\gamma_{d}}{\gamma_{1}-\gamma_{d}}t\;\exp\left(-\gamma_{1}t\right)-\frac{\gamma_{1}\gamma_{d}}{{\left(\gamma_{1}-\gamma_{d}\right)}^{2}}\exp\left(-\gamma_{1}t\right)\right)$$

$$\frac{dy_{1}}{dc_{0}^{pb}}=\alpha \;\exp\left(-\gamma_{1}t\right)$$

$$\frac{dy_{1}}{dc_{0}^{bm}}=\alpha t\exp\left(-\gamma_{1}t\right)$$

$$\frac{dy_{1}}{d\alpha }=\frac{\gamma_{1}\gamma_{d}B_{0}}{{\left(\gamma_{1}-\gamma_{d}\right)}^{2}}\exp\left(\gamma_{d}t\right)+\left(c_{0}^{bm}-\frac{\gamma_{d}B_{0}}{\gamma_{1}-\gamma_{d}}\right)t\;exp(-\gamma_{1}t)+\left(c_{0}^{pb}-\frac{\gamma_{1}\gamma_{d}B_{0}}{{\left(\gamma_{1}-\gamma_{d}\right)}^{2}}\right)exp\left(-\gamma_{1}t\right)$$

$$\frac{dy_{1}}{d\gamma_{t}}=\alpha \left(\frac{-\gamma_{d}^{2}B_{0}}{{\left(\gamma_{1}-\gamma_{d}\right)}^{3}}\exp\left(-\gamma_{d}t\right)+\left(c_{0}^{bm}t+\frac{\gamma_{1}\gamma_{d}B_{0}t^{2}}{2\left(\gamma_{1}-\gamma_{d}\right)}-\frac{1}{2}\gamma_{1}c_{0}^{bm}t^{2}\right)\exp\left(-\gamma_{1}t\right)\right)$$

$$\frac{dy_{1}}{d\gamma_{d}}=\alpha \left(\left(\frac{\gamma_{1}B_{0}}{{\left(\gamma_{1}-\gamma_{d}\right)}^{2}}-\frac{\gamma_{d}B_{0}t}{\gamma_{1}-\gamma_{d}}\right)\exp\left(-\gamma_{d}t\right)-\frac{\gamma_{1}B_{0}}{{\left(\gamma_{1}-\gamma_{d}\right)}^{2}}\exp\left(-\gamma_{1}t\right)\right)$$

$$\frac{dy_{1}}{d\gamma_{\deg}}=\alpha \left(-\frac{\gamma_{d}B_{0}}{{\left(\gamma_{1}-\gamma_{d}\right)}^{2}}\exp\left(-\gamma_{d}t\right)+\left(c_{0}^{bm}-\frac{\gamma_{d}B_{0}}{\gamma_{1}-\gamma_{d}}\right)t\;\exp\left(-\gamma_{1}t\right)\right)$$

$$if\;\gamma_{\deg}=\gamma_{d}=\gamma_{2},\gamma_{d}\neq \gamma_{t}\text{,}$$

$$\frac{dy_{1}}{dB_{0}}=\alpha \left(\frac{\gamma_{t}\gamma_{2}}{\gamma_{t}-\gamma_{2}}t\;\exp\left(-\gamma_{2}t\right)+\frac{\gamma_{2}}{{\left(\gamma_{2}-\gamma_{t}\right)}^{2}}\exp\left(-\gamma_{t}t\right)-\frac{\gamma_{2}}{{\left(\gamma_{2}-\gamma_{t}\right)}^{2}}\exp\left(-\gamma_{2}t\right)\right)$$

$$\frac{dy_{1}}{dc_{0}^{pb}}=\alpha \;\exp\left(-\gamma_{2}t\right)$$

$$\frac{dy_{1}}{dc_{0}^{bm}}=\alpha \left(\frac{1}{\gamma_{2}-\gamma_{t}}\exp\left(-\gamma_{t}t\right)-\frac{1}{\gamma_{2}-\gamma_{t}}\exp\left(-\gamma_{2}t\right)\right)$$

$$\frac{dy_{1}}{d\alpha }=\frac{\gamma_{t}\gamma_{2}B_{0}}{\gamma_{t}-\gamma_{2}}t\;\exp\left(-\gamma_{2}t\right)+\left(\frac{c_{0}^{bm}}{\gamma_{2}-\gamma_{t}}+\frac{\gamma_{2}B_{0}}{{\left(\gamma_{2}-\gamma_{t}\right)}^{2}}\right)\exp\left(-\gamma_{t}t\right)+\left(c_{0}^{pb}-\frac{c_{0}^{bm}}{\gamma_{2}-\gamma_{t}}-\frac{\gamma_{2}B_{0}}{{\left(\gamma_{2}-\gamma_{t}\right)}^{2}}\right)\exp\left(-\gamma_{2}t\right)$$

$$\frac{dy_{1}}{d\gamma_{t}}=\alpha \left(\frac{-\gamma_{2}^{2}B_{0}}{{\left(\gamma_{t}-\gamma_{2}\right)}^{2}}t\;\exp(-\gamma_{2}t)+\left(\frac{c_{0}^{bm}\gamma_{2}-\gamma_{2}\gamma_{t}B_{0}t+\gamma_{2}\gamma_{t}c_{0}^{bm}t+\gamma_{t}^{2}c_{0}^{bm}t}{{\left(\gamma_{2}-\gamma_{t}\right)}^{2}}+\frac{2\gamma_{2}\gamma_{t}B_{0}}{{\left(\gamma_{2}-\gamma_{t}\right)}^{3}}\right)exp\left(-\gamma_{t}t\right)\right)$$

$$\frac{dy_{1}}{d\gamma_{d}}=\alpha \left(\gamma_{t}B_{0}\left(\frac{\gamma_{t}t}{\gamma_{t}-\gamma_{2}}-\frac{\gamma_{2}t^{2}}{2\left(\gamma_{t}-\gamma_{2}\right)}\right)\exp\left(-\gamma_{2}t\right)+\frac{\gamma_{t}^{2}B_{0}}{{\left(\gamma_{t}-\gamma_{2}\right)}^{3}}\exp\left(-\gamma_{2}t\right)\right)$$

$$\frac{dy_{1}}{d\gamma_{\deg}}=\alpha \left(\left(-\frac{\gamma_{2}B_{0}}{{\left(\gamma_{t}-\gamma_{2}\right)}^{2}}\exp(\left(\gamma_{t}-2\gamma_{2}\right)t\right)+\left(c_{0}^{bm}-\frac{\gamma_{2}B_{0}}{\gamma_{t}-\gamma_{2}}\right)t\;\exp\left(-\gamma_{2}t\right)\right)$$

$$if\;\gamma_{\deg}\neq \gamma_{t}\neq \gamma_{d}\text{,}$$

$$\frac{dy_{1}}{dB_{0}}=\alpha \left(\frac{\gamma_{t}\gamma_{d}}{\left(\gamma_{deg}-\gamma_{d}\right)\left(\gamma_{t}-\gamma_{d}\right)}exp\left(-\gamma_{d}t\right)-\frac{\gamma_{t}\gamma_{d}}{\left(\gamma_{deg}-\gamma_{t}\right)\left(\gamma_{t}-\gamma_{d}\right)}\text{exp}\left(-\gamma_{t}t\right)+\left(\frac{\gamma_{t}\gamma_{d}}{\left(\gamma_{t}-\gamma_{d}\right)\left(\gamma_{deg}-\gamma_{d}\right)}+\frac{\gamma_{t}\gamma_{d}}{\left(\gamma_{deg}-\gamma_{t}\right)\left(\gamma_{t}-\gamma_{d}\right)}\right)\text{exp}\left(\text{-}\gamma_{deg}t\right)\right)$$

$$\frac{dy_{1}}{dc_{0}^{pb}}=\alpha \;\exp\left(-\gamma_{\deg}t\right)$$

$$\frac{dy_{1}}{dc_{0}^{bm}}=\alpha \left(\frac{\left(\gamma_{t}-\gamma_{d}\right)\gamma_{t}}{\left(\gamma_{\deg}-\gamma_{t}\right)\left(\gamma_{t}-\gamma_{d}\right)}\exp\left(-\gamma_{t}t\right)-\frac{\gamma_{t}}{\gamma_{\deg}-\gamma_{t}}\right)\exp\left(-\gamma_{\deg}t\right)$$

$$\frac{dy_{1}}{d\alpha }=\frac{\gamma_{t}\gamma_{d}B_{0}}{\left(\gamma_{\deg}-\gamma_{d}\right)\left(\gamma_{t}-\gamma_{d}\right)}\exp\left(-\gamma_{d}t\right)+\frac{\left(\gamma_{t}-\gamma_{d}\right)\gamma_{t}c_{0}^{bm}-\gamma_{\gamma }\gamma_{d}B_{0}}{\left(\gamma_{\deg}-\gamma_{t}\right)\left(\gamma_{t}-\gamma_{d}\right)}\exp\left(-\gamma_{t}t\right)+\left(c_{0}^{pb}+\frac{\gamma_{t}\gamma_{d}B_{0}}{\left(\gamma_{t}-\gamma_{d}\right)\left(\gamma_{deg}-\gamma_{d}\right)}-\frac{\gamma_{t}c_{0}^{bm}}{\gamma_{deg}-\gamma_{t}}+\frac{\gamma_{t}\gamma_{d}B_{0}}{\left(\gamma_{deg}-\gamma_{t}\right)\left(\gamma_{t}-\gamma_{d}\right)}\right)\text{exp}\left(\text{-}\gamma_{deg}t\right)$$

$$\frac{dy_{1}}{d\gamma_{t}}=\alpha \left(-\frac{\gamma_{d}^{2}B_{0}}{\left(\gamma_{\deg}-\gamma_{d}\right){\left(\gamma_{t}-\gamma_{d}\right)}^{2}}\exp(-\gamma_{d}t)+\left(\frac{c_{0}^{bm}\gamma_{t}}{{\left(\gamma_{deg}-\gamma_{t}\right)}^{2}\left(\gamma_{t}-\gamma_{d}\right)}-\frac{\gamma_{d}^{2}B_{0}}{{\left(\gamma_{t}-\gamma_{d}\right)}^{2}\left(\gamma_{deg}-\gamma_{d}\right)}+\frac{\gamma_{d}\gamma_{t}^{2}B_{0}-\gamma_{d}^{2}\gamma_{deg}B_{0}}{{\left(\gamma_{deg}-\gamma_{d}\right)}^{2}{\left(\gamma_{t}-\gamma_{d}\right)}^{2}}\right)exp\left(-\gamma_{deg}t\right)+\left(\frac{\gamma_{\deg}c_{0}^{bm}}{{\left(\gamma_{\deg}-\gamma_{t}\right)}^{2}}+\frac{\gamma_{\deg}\gamma_{d}^{2}B_{0}-\gamma_{d}B_{0}\gamma_{t}^{2}}{{\left(\gamma_{\deg}-\gamma_{t}\right)}^{2}}+\frac{\gamma_{t}^{2}c_{0}^{bm}-\gamma_{t}\gamma_{d}c_{0}^{bm}-\gamma_{t}\gamma_{d}B_{0}}{\left(\gamma_{\deg}-\gamma_{t}\right)\left(\gamma_{t}-\gamma_{d}\right)}t\right)\exp\left(-\gamma_{t}t\right)\right)$$

$$\frac{dy_{1}}{d\gamma_{d}}=\alpha \left(\left(\frac{\gamma_{\deg}\gamma_{t}^{2}B_{0}-\gamma_{d}^{2}\gamma_{t}B_{0}}{{\left(\gamma_{t}-\gamma_{d}\right)}^{2}{\left(\gamma_{\deg}-\gamma_{d}\right)}^{2}}-\frac{\gamma_{d}\gamma_{t}B_{0}t}{\left(\gamma_{\deg}-\gamma_{d}\right)\left(\gamma_{t}-\gamma_{d}\right)}\right)\exp\left(-\gamma_{d}t\right)-\frac{\gamma_{t}^{2}B_{0}}{{\left(\gamma_{\deg}-\gamma_{t}\right)\left(\gamma_{t}-\gamma_{d}\right)}^{2}}\exp\left(-\gamma_{t}t\right)+\left(\frac{\gamma_{\deg}\gamma_{t}^{2}B_{0}-\gamma_{d}^{2}\gamma_{t}B_{0}}{{\left(\gamma_{t}-\gamma_{d}\right)}^{2}{\left(\gamma_{\deg}-\gamma_{d}\right)}^{2}}+\frac{\gamma_{t}^{2}B_{0}}{{\left(\gamma_{\deg}-\gamma_{t}\right)\left(\gamma_{t}-\gamma_{d}\right)}^{2}}\right)\exp\left(-\gamma_{\deg}t\right)\right)$$

$$\frac{dy_{1}}{d\gamma_{\deg}}=\alpha \left(-\frac{\gamma_{t}\gamma_{d}B_{0}}{{\left(\gamma_{deg}-\gamma_{d}\right)}^{2}\left(\gamma_{t}-\gamma_{d}\right)}\text{exp}\;t\left(-\gamma_{d}t\right)-\frac{\gamma_{t}^{2}c_{0}^{bm}-\gamma_{d}\gamma_{t}c_{0}^{bm}-\gamma_{t}\gamma_{d}B_{0}}{{\left(\gamma_{deg}-\gamma_{t}\right)}^{2}\left(\gamma_{t}-\gamma_{d}\right)}\text{exp}\left(-\gamma_{t}t\right)+\left(-c_{0}^{pb}t-\frac{\gamma_{t}\gamma_{d}B_{0}}{\left(\gamma_{t}-\gamma_{d}\right){\left(\gamma_{deg}-\gamma_{d}\right)}^{2}}-\frac{\gamma_{t}\gamma_{d}B_{0}t}{\left(\gamma_{t}-\gamma_{d}\right)\left(\gamma_{deg}-\gamma_{d}\right)}+\frac{\gamma_{t}c_{0}^{bm}}{{\left(\gamma_{deg}-\gamma_{t}\right)}^{2}}+\frac{\gamma_{t}c_{0}^{bm}t}{\gamma_{deg}-\gamma_{t}}-\frac{\gamma_{t}\gamma_{d}B_{0}}{{\left(\gamma_{deg}-\gamma_{t}\right)}^{2}\left(\gamma_{t}-\gamma_{d}\right)}-\frac{\gamma_{t}\gamma_{d}B_{0}t}{\left(\gamma_{deg}-\gamma_{t}\right)\left(\gamma_{t}-\gamma_{d}\right)}\right)exp\left(-\gamma_{deg}t\right)\right)$$

#### Single patient parameter computation

We estimated the patient-specific parameters by optimizing the random effects for each patient respectively, while giving the population parameters β and D:

$${\hat{b}}_{k}\left(\beta ,D\right)=argmax_{b_{k}}\left(p\left({\left\{t_{jk},{\left\{{\bar{y}}_{ijk}\right\}}_{i=1}^{d_{t}}\right\}}_{j=1}^{d_{t}}|\beta ,b_{k}\right)+p\left(b_{k}|D\right)\right)\text{.}$$

Here, i,j,k is the index of measurement, time point and patient respectively, β is the fixed effects, and D is the covariance matrix for the random effects, t is the time point, and y is the measurement data. $d_{y}$ and $d_{t}$ are the dimension of measurements and time points respectively.

## Supplemental information

Document S1. Figures S1—S3 and Tables S1 and S2
