# Supplementary material for: Modeling early treatment response in AML from cell-free tumor DNA
Source: iScience. 2023 Oct 19;26(12):108271. doi: 10.1016/j.isci.2023.108271 (PMC10690559; doi:10.1016/j.isci.2023.108271)
Supplement: Document S1. Figures S1—S3 and Tables S1 and S2 [file mmc1.pdf]

iScience, Volume *26*

Supplemental information

Modeling early treatment response

in AML from cell-free tumor DNA

Dantong Wang, Christian Rausch, Simon A. Buerger, Sebastian Tschuri, Maja Rothenberg-Thurley, Melanie Schulz, Jan Hasenauer, Frank Ziemann, Klaus H. Metzeler, and Carsten Marr

Supplementary Figures & Tables

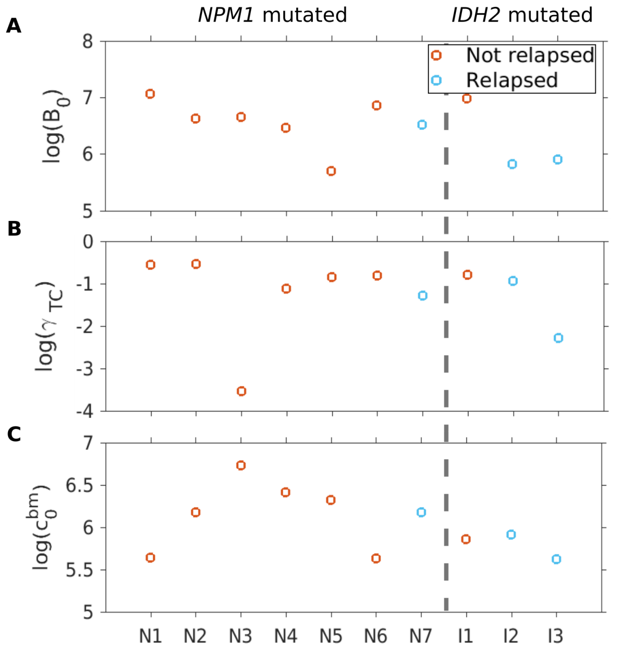

Figure S1. Parameter estimates for the individual patients. Related to Figure 5.

(A) Initial values of blast cell numbers for each patient.

(B) Death rate of blast cells in the bone marrow for each patient.

(C) Initial values of ctDNA in bone marrow compartment for each patient.

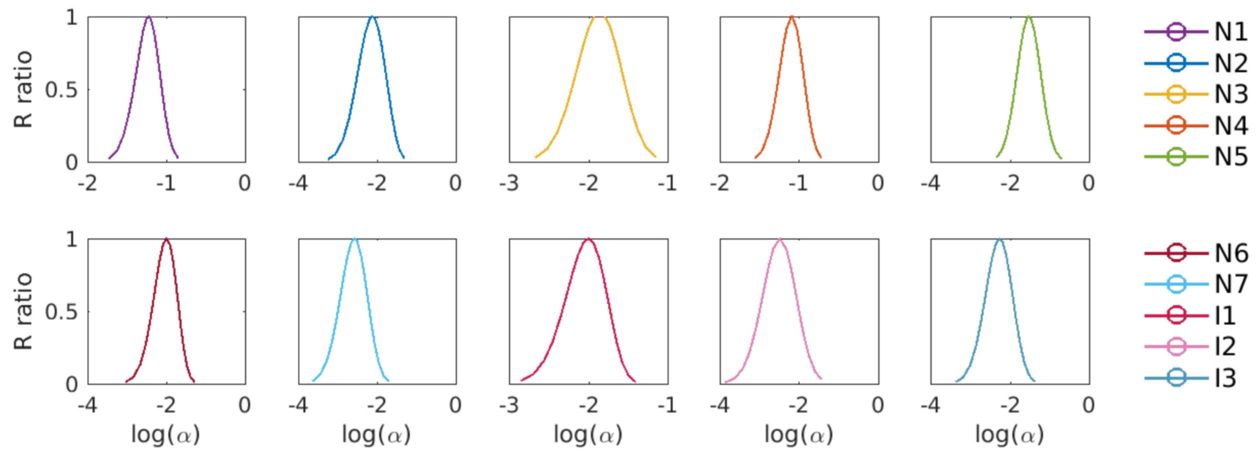

Figure S2. Profile likelihood of α values for individual patients given population parameter values. Related to Figure 5.

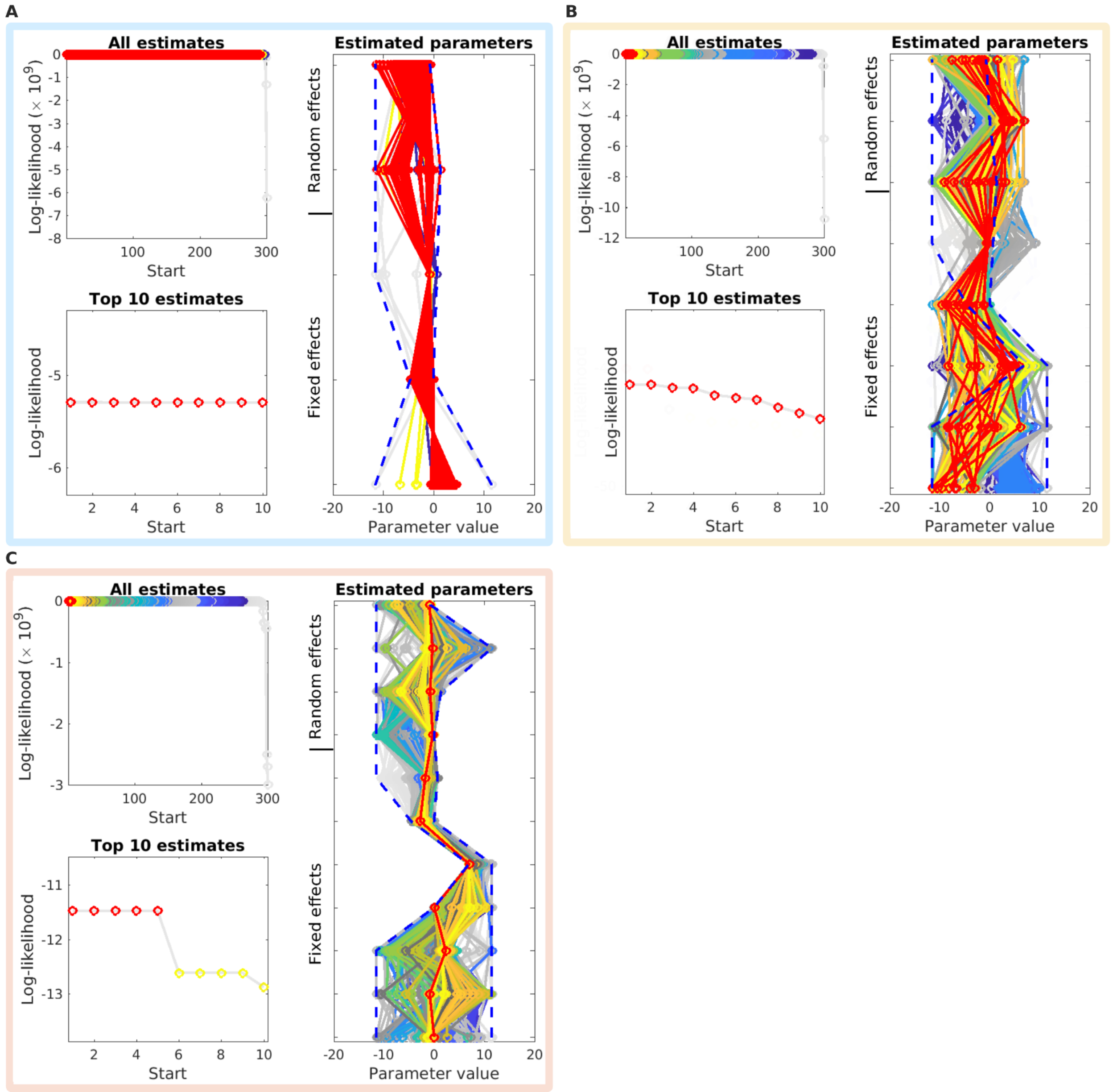

Figure S3. Waterfall plot of (A) one-step model, (B) two-step model and (C) three-step model. Related to Figure 2.

Table S1. Genetic variants of AML patients identified by targeted NGS at initial diagnosis and relapse. Related to Table 1.

Patient ID	initial diagnosis				relapse			
	**Variants with clinical significance**	**VAF (%)**	**Variants of uncertain significance (VUS)**	**VAF (%)**	**Variants with clinical significance**	**VAF (%)**	**Variants of uncertain significance (VUS)**	**VAF (%)**
**N1**	*NPM1*<br>*FLT3-ITD*<br>*TET2*<br>*TET2*	47,98<br>22,64<br>41,78<br>42,14	*ETV6*<br>*GATA2*	5,03<br>43,21				
**N2**	*NMP1*	31,81	*Kit*	49,07				
**N3**	*NPM1*<br>*FLT3*<br>*DNMT3A*	48,34<br>35,98<br>44,51	*SH2B2*<br>*ASXL1*	46,95<br>49,89				
**N4**	*NPM1*<br>*KRAS*<br>*DNMT3A*<br>*FLT3-ITD*	46,96<br>35,55<br>43,18<br>Ratio: 0.08*						
**N5**	*NPM1*<br>*NRAS*<br>*DNMT3A*<br>*JAK2*	43,24<br>44,64<br>46,03<br>47,67						
**N6**	*NPM1*<br>*DNMT3A*<br>*NRAS*	48,02<br>44,82<br>6,22	*SH2B3*	49,02				
**N7**	*NPM1*<br>*DNMT3A*<br>*TET2*<br>*TET2*<br>*FLT3-ITD*	19,02<br>11,33<br>22,51<br>37,06<br>28,88§	*PTPN11*	51,48	*NPM1*#<br>*DNMT3A*<br>*TET2*<br>*TET2*	<1,0#<br>16,85<br>11,82<br>38,41	*PTPN11*	49,29
**I1**	*IDH2*<br>*STAG2*<br>*EZH2*	40,8<br>42,11<br>44,82						
**I2**	*IDH2*<br>*CBPA*<br>*CBPA*<br>*EZH2*<br>*EZH2*<br>*ASXL1*<br>*STAG2*	41,0<br>44,0<br>46,0<br>3,0<br>42,0<br>40,0<br>88,0			*IDH2*<br>*CBPA*<br>*CBPA*<br>*EZH2*<br>*EZH2*<br>*ASXL1*<br>*STAG2*	43,88<br>45,08<br>73,27¶<br>48,7¶<br>47,15<br>50,97<br>88,4		
**I3**	*IDH2*<br>*ASXL1*<br>*SRSF2*	39,68<br>42,91<br>44,34			*IDH2*<br>*ASXL1*<br>*SRSF2*<br>*RUNX1*	42,84<br>41,99<br>40,61<br>34,96¶		

**FLT-ITD* Ratio was assessed by routine qPCR and fragment analysis
#*NPM1* positive relapse was also determined by routine qPCR-based MRD-monitoring
¶ Variants that were newly detected in relapse
§ Variants that were not detected in relapse
VAF=Variant allel frequency; VUS = Variants of uncertain significance

Table S2. Estimated parameter values of the two-step and three-step model. Related to Figure 2.

Two-step model	Parameter	B_0 x 10^9	c_0^{pb} x 10^9	γ_t	α	Var(B_0)	Var(c_0^{pb})	Var(α)
	Value	25.26	0.76	0.00033	0.00077	13.55	0.088	4.60
Three-step model	Parameter	B_0 x 10	c_0^{pb} x 10^9	γ_d	γ_t	s_c	α	Var(B_0)
	Value	1139.97	10.12	1.00	0.43	1.14	0.063	0.72
	Parameter	Var(c_0^{pb})	Var(γ_d)	Var(α)				
	Value	0.45	0.77	0.41				
